# Calsequestrin interacts directly with the cardiac ryanodine receptor luminal domain

**DOI:** 10.1242/jcs.191643

**Published:** 2016-11-01

**Authors:** Ahmed Handhle, Chloe E. Ormonde, N. Lowri Thomas, Catherine Bralesford, Alan J. Williams, F. Anthony Lai, Spyros Zissimopoulos

**Affiliations:** 1 Sir Geraint Evans Wales Heart Research Institute, School of Medicine, Cardiff University, Cardiff CF14 4XN, UK; 2 Medical Biochemistry Department, Faculty of Medicine, Mansoura University, Mansoura 35516, Egypt

**Keywords:** Ca^2+^ intracellular release, Calsequestrin, Excitation-contraction coupling, Luminal Ca^2+^ sensor, Protein–protein interaction, Ryanodine receptor, Sarcoplasmic reticulum

## Abstract

Cardiac muscle contraction requires sarcoplasmic reticulum (SR) Ca^2+^ release mediated by the quaternary complex comprising the ryanodine receptor 2 (RyR2), calsequestrin 2 (CSQ2), junctin (encoded by *ASPH*) and triadin. Here, we demonstrate that a direct interaction exists between RyR2 and CSQ2. Topologically, CSQ2 binding occurs at the first luminal loop of RyR2. Co-expression of RyR2 and CSQ2 in a human cell line devoid of the other quaternary complex proteins results in altered Ca^2+^-release dynamics compared to cells expressing RyR2 only. These findings provide a new perspective for understanding the SR luminal Ca^2+^ sensor and its involvement in cardiac physiology and disease.

## Abstract (precis)

**Summary:** Direct physical and functional calsequestrin 2 association with the ryanodine receptor luminal domain is the minimal requirement to form the sarcoplasmic reticulum Ca^2+^ sensor, which governs cardiac Ca^2+^ homeostasis and reliable heartbeat.

## INTRODUCTION

Cardiac excitation–contraction coupling, where an action potential elicits cardiomyocyte contraction, is mediated by ‘Ca^2+^-induced Ca^2+^ release’. Upon plasma membrane depolarisation, Ca^2+^ influx through voltage-gated Ca^2+^ channels triggers Ca^2+^ release through the sarcoplasmic reticulum (SR)-located RyR2 to increase cytoplasmic Ca^2+^ concentration and transduce sarcomere contraction (Bers, 2002). Termination of SR Ca^2+^ release is an incompletely understood process but appears to involve intra-SR luminal Ca^2+^ regulation of RyR2 channel activity (Radwański et al., 2013; Terentyev et al., 2002). Luminal Ca^2+^ exerts its effects directly on RyR2 (Chen et al., 2014) and through the cardiac SR Ca^2+^-binding protein CSQ2 (Beard et al., 2009; Gaburjakova et al., 2013; Gyorke and Terentyev, 2008). Abnormal RyR2 regulation by luminal Ca^2+^ causes diastolic SR Ca^2+^ leak, resulting in arrhythmias and heart failure (Gyorke and Terentyev, 2008; Priori and Chen, 2011). Catecholaminergic polymorphic ventricular tachycardia (CPVT) is an outstanding example of how arrhythmia induces sudden cardiac death by perturbing Ca^2+^ handling (Cerrone et al., 2009). CPVT type 1 is linked to RyR2 mutations inherited in an autosomal-dominant manner, whereas CPVT type 2 is linked to both dominant and recessive mutations of calsequestrin 2 (CSQ2) (http://triad.fsm.it/cardmoc/).

RyR2 exists as a macromolecular complex composed of CSQ2 and the SR integral membrane proteins triadin and junctin (encoded by *ASPH*), which together form the luminal Ca^2+^ sensor (Zhang et al., 1997). CSQ2 interacts directly with both triadin and junctin in a Ca^2+^-dependent manner, with 1–5 mM Ca^2+^ inhibiting these interactions (Shin et al., 2000; Zhang et al., 1997). Their binding sites have been mapped to the Asp-rich region at the C-terminus of CSQ2 and to the KEKE motif (residues 200–224) of triadin (Kobayashi et al., 2000; Shin et al., 2000). Triadin and junctin interactions with RyR1 and RyR2 are also known to be direct, with the RyR2–junctin interaction being Ca^2+^-independent (Guo and Campbell, 1995; Zhang et al., 1997). The triadin KEKE motif interacts with negatively charged residues within the second luminal loop of RyR1 (Goonasekera et al., 2007; Lee et al., 2004), whereas junctin association with the RyR1 or RyR2 involves both luminal and cytoplasmic sites (Altschafl et al., 2011; Li et al., 2015). Evidence for direct RyR–CSQ association is very scarce. Interaction of calsequestrin 1 (CSQ1) with purified native skeletal muscle RyR1 has been reported (Herzog et al., 2000), which could have differential effects on channel activity depending on the presence of triadin and/or junctin (Beard et al., 2002; Szegedi et al., 1999). To our knowledge, there is no biochemical data for direct physical association between RyR2 and CSQ2 in the heart. Thus, although the functional association of RyR2 and CSQ2 is well established, this is generally believed to be indirectly mediated through triadin and junctin interactions (Beard et al., 2009; Gaburjakova et al., 2013; Gyorke and Terentyev, 2008).

## RESULTS AND DISCUSSION

### Direct association of calsequestrin 2 with RyR2

**Fig. 1.**
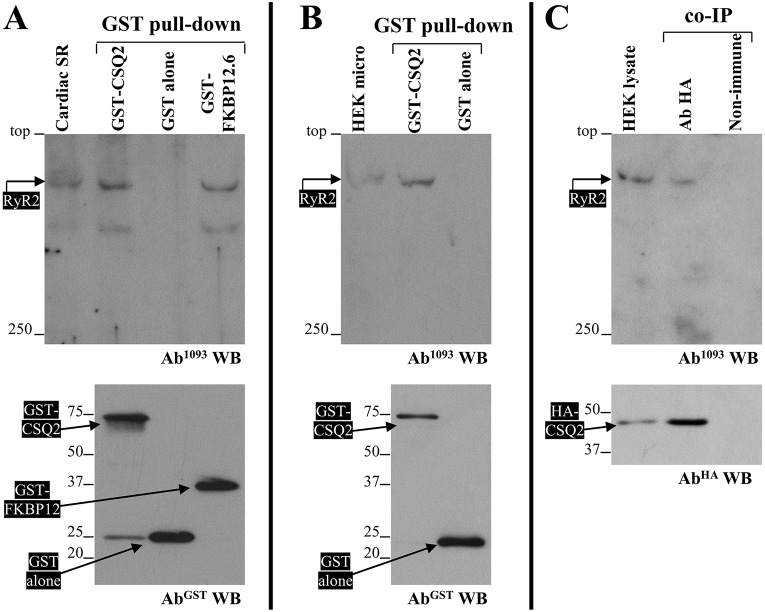
**CSQ2 displays a direct interaction with RyR2.** (A) GST pull-down experiments (*n*=3) of native RyR2 incubated with purified GST-fusion proteins as indicated. Solubilised pig cardiac SR vesicles were incubated with purified GST-fusion proteins that had been captured on glutathione beads. Beads were precipitated by centrifugation, and the presence of associated RyR2 was analysed by SDS-PAGE (4% gel) and immunoblotting using the Ab^1093^ antibody (top). As negative control, pull-down assays were performed with GST alone. To detect the isolated GST-fusion proteins, one-tenth of pull-down samples was analysed by SDS-PAGE (12% gel) and immunoblotting using an antibody against GST (Ab^GST^, bottom). An aliquot of solubilised pig cardiac SR corresponding to 1% (5 μg) of the amount processed in the pull-down assay was also included in the gels. As a negative control, pulldown assays were performed with GST alone; GST–FKBP12.6 was used as positive control for RyR2 interaction. (B) GST pull-down experiments (*n*=3) of recombinant human RyR2 incubated with purified GST-fusion proteins as indicated. Solubilised HEK293 microsomes expressing RyR2 (HEK micro) were incubated with purified GST-fusion proteins captured on glutathione beads, and analysed as described in A above. (C) Co-immunoprecipitation (co-IP) analyses (*n*=3) of recombinant human RyR2 co-expressed with CSQ2 in mammalian HEK293 cells. HA–CSQ2 was immunoprecipitated with an antibody against HA (Ab^HA^) from solubilised HEK293 lysates, and the presence of associated RyR2 was analysed by SDS-PAGE (4% gel) and immunoblotting using Ab^1093^ (top). As negative control, co-immunoprecipitation assays were performed with non-immune rabbit IgG (Non-immune). To detect isolated HA–CSQ2, one-tenth of the immunoprecipitate was analysed by SDS-PAGE (12% gel) and western blotting (WB) using an antibody against HA (Ab^HA^, bottom). An aliquot of HEK293 cell lysate corresponding to 1% (20 μg) of the amount processed in the co-immunoprecipitation assay was also included in the gels.

Initially, we investigated the native RyR2 interaction with recombinant purified CSQ2 using GST pull-down assays. GST alone was used as negative control and GST–FKBP12.6 (also known as FKBP1B) was used as a positive control. Purified (1 µM) GST-fusion protein was incubated with CHAPS-solubilised RyR2 from pig cardiac SR and isolated with glutathione Sepharose (Fig. 1A, bottom); the presence of co-precipitated RyR2 was analysed by immunoblotting using Ab^1093^ (Fig. 1A, top). RyR2 was efficiently co-precipitated by GST–CSQ2 and GST–FKBP12.6 but not by GST alone. The interaction of recombinant GST–CSQ2 with pig cardiac RyR2 might be direct or be mediated through the native-RyR2-associated proteins triadin, junctin and calsequestrin 2. To exclude an indirect interaction, we used recombinant human RyR2 expressed in HEK293 cells – a non-cardiac cell line lacking endogenous junctin, triadin and CSQ2, as revealed by immunoblot analysis (Fig. S1). Using GST pull-down assays, we found strong GST–CSQ2 binding to recombinant human RyR2 (Fig. 1B), indicating that triadin and junctin are non-essential for the RyR2–CSQ2 association. These experiments made use of purified CSQ2 expressed in bacteria that lack the machinery for glycosylation and phosphorylation, which are potential post-translational modifications for native calsequestrin 2. We therefore assessed the RyR2–CSQ2 interaction by performing co-immunoprecipitation assays following their co-expression in mammalian HEK293 cells. Hemagglutinin (HA)-tagged CSQ2 was immunoprecipitated with an antibody against HA from CHAPS-solubilised HEK293 cell lysates (Fig. 1C, bottom), and the presence of co-precipitated RyR2 was analysed by immunoblotting using Ab^1093^ (Fig. 1C, top). RyR2 was recovered from the anti-HA immunoprecipitate but not from the negative control using non-immune rabbit IgG. In the reverse experiment, immunoprecipitation of RyR2 with Ab^1093^ (Fig. S2, bottom) resulted in co-precipitation of HA–CSQ2 (Fig. S2, top), providing further evidence for direct complex formation between RyR2 and CSQ2. Our findings are consistent with previous work that indicates a physical association between the skeletal muscle isoforms, RyR1 and CSQ1, and that made use of purified native RyR1 with no detectable levels of endogenous triadin or junctin (Beard et al., 2002; Herzog et al., 2000; Szegedi et al., 1999).

### CSQ2 interacts with the first luminal loop of RyR2

**Fig. 2.**
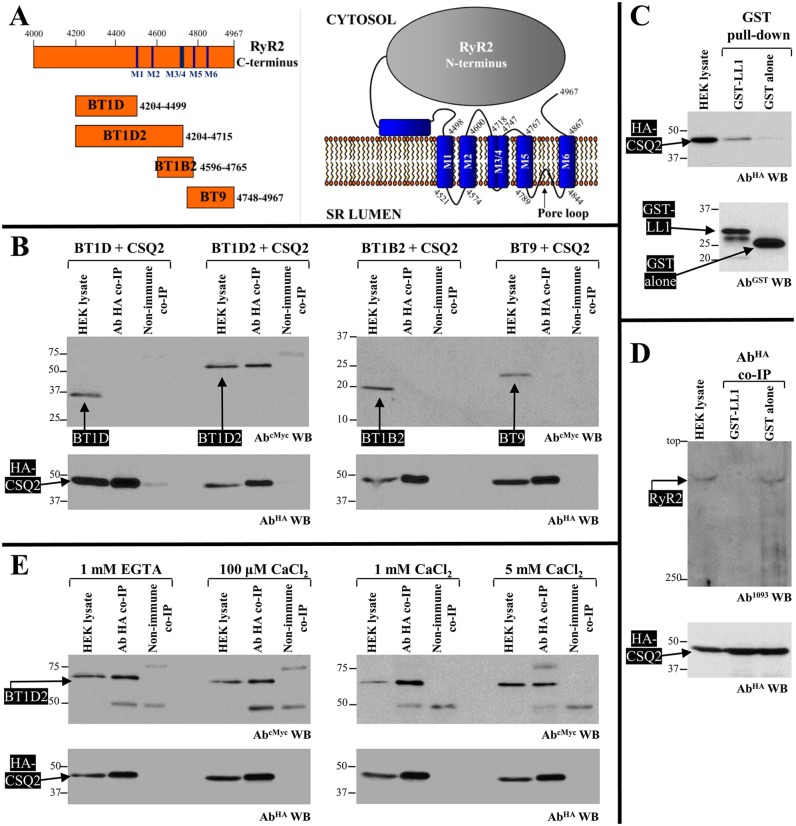
**Mapping the CSQ2-interacting site on RyR2.** (A) Human RyR2 SR membrane topology (right) and diagram of overlapping BT1D, BT1D2, BT1B2 and BT9 constructs covering the RyR2 C-terminus containing all the transmembrane domains and intervening luminal loops. M indicates membrane-spanning domains. (B) Co-immunoprecipitation (co-IP) experiments (*n*=4) of recombinant human RyR2 fragments co-expressed with CSQ2 in HEK293 cells. HA–CSQ2 was immunoprecipitated with an antibody against HA (Ab^HA^) from solubilised HEK293 lysates that co-expressed Myc-tagged BT constructs. As negative control, co-immunoprecipitation assays were performed with non-immune rabbit IgG (non-immune). Immunoprecipitated proteins were resolved in separate SDS-PAGE gels (12% for BT1D and BT1D2, 15% for BT1B2 and BT9, and 12% for HA–CSQ2) loaded with nine-tenths or one-tenth of the immunoprecipitates and were analysed by immunoblotting using antibodies against Myc (Ab^cMyc^, top) or HA (Ab^HA^, bottom), respectively. An aliquot of HEK293 cell lysate corresponding to 1% (20 μg) of the amount processed in the co-immunoprecipitation assay was also included in the gels. (C) GST pull-down experiments (*n*=4) of recombinant human CSQ2 incubated with purified GST–LL1 (RyR2 residues 4521–4573) or GST alone serving as negative control. Solubilised HEK293 lysates expressing HA–CSQ2 were incubated with purified GST-fusion proteins captured on glutathione beads. Precipitated proteins were resolved in separate 12% SDS-PAGE gels loaded with nine-tenths or one-tenth of the pull-down samples and were analysed by immunoblotting for HA (Ab^HA^, top) or GST (Ab^GST^, bottom), respectively. (D) Competition binding experiments (*n*=3) to test the RyR2–CSQ2 interaction. HA–CSQ2 was immunoprecipitated with an antibody against HA (Ab^HA^) from solubilised HEK293 lysates co-expressing RyR2 in the presence of GST–LL1 or GST alone (negative control). Immunoprecipitated proteins were resolved in separate 4% or 12% SDS-PAGE gels loaded with nine-tenths or one-tenth of the immunoprecipitates and were analysed by immunoblotting with Ab^1093^ (top) and for HA (Ab^HA^, bottom), respectively. (E) Co-immunoprecipitation experiments (*n*=4) of BT1D2 co-expressed with CSQ2 in HEK293 cells, performed under different Ca^2+^ concentrations. HA–CSQ2 was immunoprecipitated with an antibody against HA (Ab^HA^) from solubilised HEK293 lysates containing either 1 mM EGTA, 100 µM CaCl_2_, 1 mM CaCl_2_ or 5 mM CaCl_2_, as indicated. The presence of co-precipitated BT1D2 was analysed by SDS-PAGE (10% gels) and immunoblotting for Myc (Ab^cMyc^, top) or HA (Ab^HA^, bottom), respectively.

The direct interaction of CSQ2 can only occur with RyR2 peptide sequences that face the SR lumen. To map the CSQ2-binding site, we used four overlapping fragments covering the RyR2 C-terminus containing all the transmembrane domains and intervening luminal loops (BT constructs shown in Fig. 2A). HA–CSQ2, co-expressed with Myc-tagged BT1D, BT1D2, BT1B2 and BT9 constructs (see Fig. 2A) in HEK293 cells, was immunoprecipitated with an antibody against HA from CHAPS-solubilised cell lysates (Fig. 2B, bottom), and the presence of co-precipitated BT constructs was analysed by immunoblotting using an antibody against Myc (Fig. 2B, top). BT1D2 was the only RyR2 fragment recovered in the anti-HA immunoprecipitate but not in the negative control using non-immune rabbit IgG. The shorter BT1D construct that lacks the first two transmembrane domains and intervening luminal loop was found to be negative for an interaction with CSQ2. To further test whether the CSQ2-binding site is contained within the RyR2 first luminal loop (LL1), we generated the RyR2 LL1 fragment (residues 4521–4573) as a GST-fusion protein (GST-LL1) purified from bacteria for use in pull-down assays; we were unable to use the LL1 construct in coimmunoprecipitation assays because it was expressed very poorly in HEK293 cells. Purified (1 µM) GST-fusion protein was incubated with CHAPS-solubilised HEK293 cell lysate expressing HA–CSQ2 and isolated with glutathione Sepharose (Fig. 2C, bottom); the presence of co-precipitated HA–CSQ2 was analysed by immunoblotting using an antibody against HA (Fig. 2C, top). HA–CSQ2 was efficiently pulled down by GST–LL1, whereas binding to GST alone, serving as negative control, was negligible. To further assess whether the CSQ2-binding site is contained within the RyR2 first luminal loop, we conducted competition binding experiments to test whether GST–LL1 disrupted the CSQ2 interaction with the full-length RyR2. RyR2 was co-expressed with HA–CSQ2 in HEK293 cells, and co-immunoprecipitation assays were performed in the presence of 1 µM GST–LL1 or GST alone. As seen in Fig. 2D, in the presence of GST alone, RyR2 was efficiently recovered in the anti-HA immunoprecipitate, whereas the inclusion of GST–LL1 abolished the RyR2 interaction with HA–CSQ2.

The experiments described above were conducted using assay buffers containing residual, micromolar Ca^2+^. Further investigation of the Ca^2+^ sensitivity of the RyR2–CSQ2 interaction was performed in the presence of near-physiological SR Ca^2+^ concentrations (1 mM CaCl_2_), or in the absence of Ca^2+^ (1 mM EGTA), as well as under conditions of high (5 mM CaCl_2_) or low (100 µM CaCl_2_) SR Ca^2+^ load. Using co-immunoprecipitation assays, we found comparable amounts of BT1D2 binding to HA–CSQ2 in the presence of 1 mM EGTA, and 100 µM and 1 mM CaCl_2_; however, recovery of BT1D2 in the anti-HA immunoprecipitate was diminished with 5 mM CaCl_2_ (Fig. 2E). Quantitative data (*n*=4) following densitometry analysis and normalisation for the amount of protein expressed in the cell lysate indicate equivalent amounts of HA–CSQ2 binding to BT1D2 for Ca^2+^ concentrations up to 1 mM (1 mM EGTA, 154.2±20.8; 100 µM CaCl_2_, 126.7±30.5; 1 mM CaCl_2_, 149.0±43.5; mean±s.e.m.); however, 5 mM CaCl_2_ substantially reduced binding by >50% (71.5±36.9, *P*<0.05 two-tailed paired Student's *t*-test compared to 1 mM CaCl_2_). These results suggest that at Ca^2+^ levels within the physiological SR range (≤1 mM), the RyR2–CSQ2 interaction remains intact but that it is diminished at supra-millimolar Ca^2+^ levels, in agreement with previous reports (Beard et al., 2009; Gaburjakova et al., 2013). Our data are also consistent with recent single-channel recordings demonstrating that CSQ2 effects on RyR2 activity are unaffected by luminal Ca^2+^ concentration changes up to 1 mM (Chen et al., 2013; Tencerová et al., 2012). CSQ2 undergoes structural changes upon Ca^2+^ binding and polymerises at >1 mM Ca^2+^ concentrations; it is believed that a front-to-front CSQ2 dimer is first formed, with these dimers forming a back-to-back stack to produce a polymeric structure (Gaburjakova et al., 2013). Our findings suggest that the RyR2-interacting site on CSQ2 assumes a Ca^2+^-independent conformation that might be obstructed if CSQ2 is in its polymeric form. They further indicate that monomeric CSQ2 is sufficient for direct RyR2 interaction.

### Direct CSQ2 association with RyR2 regulates cellular Ca^2+^- release dynamics

**Fig. 3.**
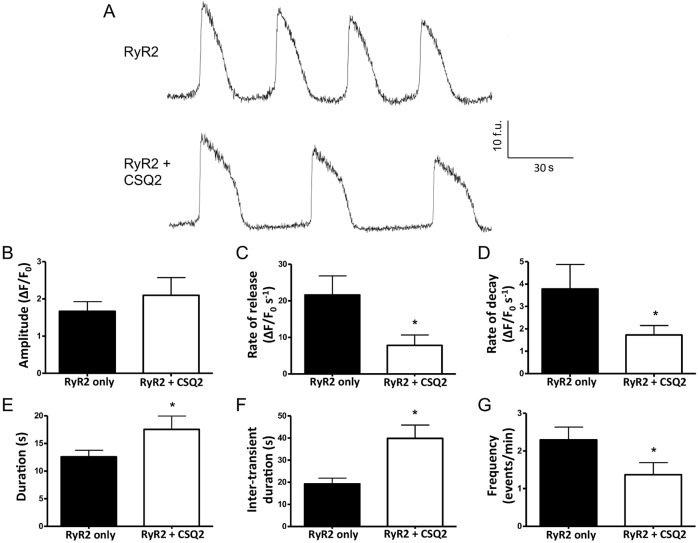
**Direct interaction of CSQ2 with RyR2 alters intracellular Ca^2+^ release.** Traces from Fluo-3-AM-loaded single HEK293 cells expressing RyR2 alone or RyR2 together with CSQ2, showing spontaneous Ca^2+^ release under conditions with 1.3 mM extracellular Ca^2^^+^ (A). Parameters of Ca^2+^-release events, including amplitude (B), rate of release (C), rate of decay (D), duration (E), inter-transient duration (F) and frequency (G) were analysed for *n*=18– 24 cells from three or four separate experiments. *Significant difference by unpaired Student's *t*-test, where *P*<0.005. Data are means±s.e.m. F denotes fluorescence; f.u. denotes fluorescence units.

Expression of RyR2 in HEK293 cells is known to result in spontaneous global Ca^2+^-release events (Jiang et al., 2002; Seidel et al., 2015). To assess the functional impact of CSQ2 on RyR2 in the absence of triadin and junctin, we generated HEK293 cells that stably expressed CSQ2, verified by immunoblotting (Fig. S1). Co-expression of RyR2 in the CSQ2-expressing stable cell line resulted in an increase in the duration between Ca^2+^-release events (inter-transient duration), decreasing their overall frequency (Fig. 3A,F,G), compared to that in cells that expressed RyR2 alone. Rates of transient rise and decay were also slower (Fig. 3C,D), lengthening the overall duration of Ca^2+^ release (Fig. 3E), with no significant change in amplitude (Fig. 3B). These whole-cell functional results provide further evidence for a direct RyR2–CSQ2 interaction and corroborate our biochemical findings. Notably, our results are consistent with the previously reported inhibitory action of this luminal accessory protein in cardiomyocytes, where CSQ2 would associate with RyR2 both directly and indirectly through junctin and triadin. The decrease in Ca^2+^-release event frequency (Fig. 3G) that we observed agrees with that reported in most CSQ2-overexpression studies (Jones et al., 1998; Sato et al., 1998; Terentyev et al., 2003; Wang et al., 2000). The longer inter-transient duration (Fig. 3F) is also consistent with the increase in Ca^2+^-binding sites within the store provided by CSQ2, indicating that more time is required to reach a high-enough level of free luminal Ca^2+^ to trigger the next Ca^2+^-release event (Jiang et al., 2002). In addition, it has been reported that although RyR2 activity decreases when CSQ2 is dissociated (Wei et al., 2009), the relative open probability increases more steeply with luminal Ca^2+^ after CSQ2 dissociation (Dulhunty et al., 2012), in agreement with our findings that the rate of spontaneous Ca^2+^ release is increased in the absence of CSQ2 (Fig. 3C). Thus, although we cannot exclude the involvement of junctin and triadin, our data obtained in a cell line devoid of these accessory proteins suggest that the functional effects of CSQ2 are mediated primarily through its direct structural interaction with RyR2.

In conclusion, our data indicate a direct functional RyR2 interaction with CSQ2, shedding more light on the quaternary structural assembly of the SR luminal Ca^2+^ sensor. Elucidation of the CSQ2-binding site within the first luminal loop of RyR2 should help in further understanding the pathologic mechanism(s) underlying arrhythmias and sudden cardiac death.

## MATERIALS AND METHODS

### Materials

Cell culture reagents and Fluo-3 AM were obtained from Life Technologies, electrophoresis equipment and reagents from Bio-Rad, protease inhibitor cocktail (Complete) from Roche, CHAPS from Merck, nProtein-A Sepharose and glutathione Sepharose 4B from GE Healthcare, secondary antibodies from Santa Cruz Biotechnology, enhanced chemiluminescence reagents from Pierce, DNA restriction endonucleases from New England Biolabs, Pfu DNA polymerase from Promega, oligonucleotides and all other reagents were from Sigma. Antibodies used for immunoprecipitation: (1 μg) rabbit anti-HA antibody (Y-11, cat. no. sc-805, 1:40 dilution for immunoprecipitation) and non-immune rabbit IgG were from Santa Cruz Biotechnology. Antibodies used in immunoblotting were mouse anti-Myc (9E10, cat. no. sc-40, 1:500 dilution for western blotting) from Santa Cruz Biotechnology, mouse anti-HA (16B12, cat. no. MMS-101, 1:1000 dilution for western blotting) from Covance; rabbit anti-GST (1:2000) (Zissimopoulos et al., 2012, 2006), and rabbit RyR2-specific (Ab^1093^, epitope at residues 4454-4474; 1:500) (Zissimopoulos et al., 2012, 2006) antibodies.

### Plasmid construction

Human CSQ2 cDNA containing the entire open reading was generated by performing PCR amplification from a human heart cDNA library (Clontech) and was cloned into the bacterial expression vector pGEX6P1 (GE Healthcare). For transient mammalian expression, CSQ2 cDNA was subcloned into a modified pCR3 vector (Life Technologies) containing an N-terminal HA peptide epitope tag, whereas for stable expression, it was cloned into pcDNA3.1^(+)^hygro (Life Technologies). Human RyR2 constructs were generated by performing PCR amplification [using human (h)RyR2 cDNA as template] and cloning into a modified pCR3 vector containing an N-terminal Myc peptide epitope tag. For bacterial expression of the LL1 construct, the cDNA was subcloned into the pGEX6P1 vector. Oligonucleotide primers and restriction enzyme cloning sites used are available upon request. All plasmids were verified by direct DNA sequencing (BigDye, Perkin-Elmer).

### Mammalian cell culture and transfection

HEK293 cells (ATCC^®^) were cultured in Dulbecco's modified Eagle's medium supplemented with 2 mM glutamine and 10% fetal bovine serum under a humidified atmosphere of 5% CO_2_ at 37°C. On reaching ∼70% confluence, cells growing on a 100-mm Petri dish were transiently transfected with 24 μg of plasmid DNA using the calcium phosphate precipitation method, as described elsewhere (Stanczyk et al., 2016). Cells were collected 24 h post-transfection and stored at −80°C. Transfected cells were also used to generate a clonally-derived CSQ2 stable cell line using limiting dilution with selection using hygromycin (400 µg ml^−1^). For Ca^2+^ imaging, stable cells from this clonal population were transiently transfected with eGFP–hRyR2 using Effectene^®^ (Qiagen) according to the manufacturer's instructions.

### Single cell Ca^2+^-imaging

Cells stably expressing CSQ2 (∼1×10^5^) were seeded onto poly-lysine-coated glass-bottomed dishes (MatTek™) before transient transfection with eGFP–hRyR2. After 48 h, cells were loaded with Fluo-3 AM (10 µM) for 45 min at 30°C. Following de-esterification, cells were immersed in Krebs-Ringer-Hepes buffer for imaging (120 mM NaCl, 5.5 mM glucose, 25 mM Hepes, 4.8 mM KCl, 1.2 mM KH_2_PO_4_, 1.2 mM MgSO_4_, 1.3 mM CaCl_2_, pH 7.4). RyR2-mediated spontaneous Ca^2+^-release events were monitored using a laser scanning confocal microscope (Leica SP5) with an oil immersion 63× objective lens with excitation at 488 nm, and Ca^2+^-dependent fluorescence emission was detected over a 520±28 nm range. Using Leica Microsystems software (LAS-AF), Ca^2+^-release events were recorded for 2 min (five frames per second) at 512×512 pixel resolution, and acquired regions of interest representing global Ca^2+^ environments (typically ∼50 μm^2^) were selected. Amplitude and temporal characteristics of spontaneous Ca^2+^ release were quantified and are displayed as means±s.e.m. Statistical analysis was performed using unpaired Student's *t*-test.

### Other methods

Bacterial expression and purification of GST-fusion proteins, GST pull-down and co-immunoprecipitation assays, SR preparation and immunoblotting were performed as described previously (Stanczyk et al., 2016; Zissimopoulos et al., 2012, 2006). Densitometry analysis was performed using a GS-700 scanner (Bio-Rad) and Quantity One software (Bio-Rad); data are expressed as means±s.e.m. and statistical analysis was performed using paired Student's *t*-test.

## References

[JCS191643C1] Altschafl B. A., Arvanitis D. A., Fuentes O., Yuan Q., Kranias E. G. and Valdivia H. H. (2011). Dual role of junctin in the regulation of ryanodine receptors and calcium release in cardiac ventricular myocytes. *J. Physiol.* 589, 6063-6080. 10.1113/jphysiol.2011.215988 22025663 PMC3286686

[JCS191643C2] Beard N. A., Sakowska M. M., Dulhunty A. F. and Laver D. R. (2002). Calsequestrin is an inhibitor of skeletal muscle ryanodine receptor calcium release channels. *Biophys. J.* 82, 310-320. 10.1016/S0006-3495(02)75396-4 11751318 PMC1302471

[JCS191643C3] Beard N. A., Wei L. and Dulhunty A. F. (2009). Ca(2+) signaling in striated muscle: the elusive roles of triadin, junctin, and calsequestrin. *Eur. Biophys. J.* 39, 27-36. 10.1007/s00249-009-0449-6 19434403

[JCS191643C4] Bers D. (2002). Cardiac excitation–contraction coupling. *Nature* 415, 198-205. 10.1038/415198a 11805843

[JCS191643C5] Cerrone M., Napolitano C. and Priori S. G. (2009). Catecholaminergic polymorphic ventricular tachycardia: a paradigm to understand mechanisms of arrhythmias associated to impaired Ca(2+) regulation. *Heart Rhythm* 6, 1652-1659. 10.1016/j.hrthm.2009.06.033 19879546

[JCS191643C6] Chen H., Valle G., Furlan S., Nani A., Gyorke S., Fill M. and Volpe P. (2013). Mechanism of calsequestrin regulation of single cardiac ryanodine receptor in normal and pathological conditions. *J. Gen. Physiol.* 142, 127-136. 10.1085/jgp.201311022 23858002 PMC3727306

[JCS191643C7] Chen W., Wang R., Chen B., Zhong X., Kong H., Bai Y., Zhou Q., Xie C., Zhang J., Guo A. et al. (2014). The ryanodine receptor store-sensing gate controls Ca2+ waves and Ca2+-triggered arrhythmias. *Nat. Med.* 20, 184-192. 10.1038/nm.3440 24441828 PMC4269524

[JCS191643C8] Dulhunty A. F., Wium E., Li L., Hanna A. D., Mirza S., Talukder S., Ghazali N. A. A. and Beard N. A. (2012). Proteins within the intracellular calcium store determine cardiac RyR channel activity and cardiac output. *Clin. Exp. Pharmacol. Physiol.* 39, 477-484. 10.1111/j.1440-1681.2012.05704.x 22524859

[JCS191643C9] Gaburjakova M., Bal N. C., Gaburjakova J. and Periasamy M. (2013). Functional interaction between calsequestrin and ryanodine receptor in the heart. *Cell. Mol. Life Sci.* 70, 2935-2945. 10.1007/s00018-012-1199-7 23109100 PMC11113811

[JCS191643C10] Goonasekera S. A., Beard N. A., Groom L., Kimura T., Lyfenko A. D., Rosenfeld A., Marty I., Dulhunty A. F. and Dirksen R. T. (2007). Triadin binding to the C-terminal luminal loop of the ryanodine receptor is important for skeletal muscle excitation–contraction coupling. *J. Gen. Physiol.* 130, 365-378. 10.1085/jgp.200709790 17846166 PMC2151650

[JCS191643C11] Guo W. and Campbell K. (1995). Association of triadin with the ryanodine receptor and calsequestrin in the lumen of the sarcoplasmic reticulum. *J. Biol. Chem.* 270, 9027-9030. 10.1074/jbc.270.16.9027 7721813

[JCS191643C12] Gyorke S. and Terentyev D. (2008). Modulation of ryanodine receptor by luminal calcium and accessory proteins in health and cardiac disease. *Cardiovasc. Res.* 77, 245-255. 10.1093/cvr/cvm038 18006456

[JCS191643C13] Herzog A., Szegedi C., Jona I., Herberg F. W. and Varsanyi M. (2000). Surface plasmon resonance studies prove the interaction of skeletal muscle sarcoplasmic reticular Ca^2+^ release channel/ryanodine receptor with calsequestrin. *FEBS Lett.* 472, 73-77. 10.1016/S0014-5793(00)01431-9 10781808

[JCS191643C14] Jiang D., Xiao B., Zhang L. and Chen S. R. W. (2002). Enhanced basal activity of a cardiac Ca^2+^ release channel (ryanodine receptor) mutant associated with ventricular tachycardia and sudden death. *Circ. Res.* 91, 218-225. 10.1161/01.RES.0000028455.36940.5E 12169647

[JCS191643C15] Jones L. R., Suzuki Y. J., Wang W., Kobayashi Y. M., Ramesh V., Franzini-Armstrong C., Cleemann L. and Morad M. (1998). Regulation of Ca^2+^ signaling in transgenic mouse cardiac myocytes overexpressing calsequestrin. *J. Clin. Invest.* 101, 1385-1393. 10.1172/JCI1362 9525981 PMC508716

[JCS191643C16] Kobayashi Y., Alseikhan B. and Jones L. (2000). Localization and characterization of the calsequestrin-binding domain of triadin 1. Evidence for a charged beta-strand in mediating the protein-protein interaction. *J. Biol. Chem.* 275, 17639-17646. 10748065 10.1074/jbc.M002091200

[JCS191643C17] Lee J., Rho S.-H., Shin D., Cho C., Park W., Eom S., Ma J. and Kim D. (2004). Negatively charged amino acids within the intraluminal loop of ryanodine receptor are involved in the interaction with triadin. *J. Biol. Chem.* 279, 6994-7000. 10.1074/jbc.M312446200 14638677

[JCS191643C18] Li L., Mirza S., Richardson S. J., Gallant E. M., Thekkedam C., Pace S. M., Zorzato F., Liu D., Beard N. A. and Dulhunty A. F. (2015). A new cytoplasmic interaction between junctin and ryanodine receptor Ca2+ release channels. *J. Cell Sci.* 128, 951-963. 10.1242/jcs.160689 25609705 PMC4342579

[JCS191643C19] Priori S. G. and Chen S. R. W. (2011). Inherited dysfunction of sarcoplasmic reticulum Ca^2+^ handling and arrhythmogenesis. *Circ. Res.* 108, 871-883. 10.1161/CIRCRESAHA.110.226845 21454795 PMC3085083

[JCS191643C20] Radwański P. B., Belevych A. E., Brunello L., Carnes C. A. and Györke S. (2013). Store-dependent deactivation: cooling the chain-reaction of myocardial calcium signaling. *J. Mol. Cell. Cardiol.* 58, 77-83. 10.1016/j.yjmcc.2012.10.008 23108187 PMC4068615

[JCS191643C21] Sato Y., Ferguson D. G., Sako H., Dorn G. W., Kadambi V. J., Yatani A., Hoit B. D., Walsh R. A. and Kranias E. G. (1998). Cardiac-specific overexpression of mouse cardiac calsequestrin is associated with depressed cardiovascular function and hypertrophy in transgenic mice. *J. Biol. Chem.* 273, 28470-28477. 10.1074/jbc.273.43.28470 9774476

[JCS191643C22] Seidel M., Thomas N. L., Williams A. J., Lai F. A. and Zissimopoulos S. (2015). Dantrolene rescues aberrant N-terminus intersubunit interactions in mutant pro-arrhythmic cardiac ryanodine receptors. *Cardiovasc. Res.* 105, 118-128. 10.1093/cvr/cvu240 25411383

[JCS191643C23] Shin D. W., Ma J. and Kim D. H. (2000). The asp-rich region at the carboxyl-terminus of calsequestrin binds to Ca^2+^ and interacts with triadin. *FEBS Lett.* 486, 178-182. 10.1016/S0014-5793(00)02246-8 11113462

[JCS191643C24] Stanczyk P. J., Lai F. A. and Zissimopoulos S. (2016). Genetic and biochemical approaches for in vivo and in vitro assessment of protein oligomerization: the ryanodine receptor case study. *J. Vis. Exp.* 113, e54271 10.3791/54271 PMC5065051 27500320

[JCS191643C25] Szegedi C., Sárközi S., Herzog A., Jóna I. and Varsányi M. (1999). Calsequestrin: more than ‘only’ a luminal Ca^2+^ buffer inside the sarcoplasmic reticulum. *Biochem. J.* 337, 19-22. 10.1042/bj3370019 9854019 PMC1219930

[JCS191643C26] Tencerová B., Zahradníková A., Gaburjáková J. and Gaburjáková M. (2012). Luminal Ca2+ controls activation of the cardiac ryanodine receptor by ATP. *J. Gen. Physiol.* 140, 93-108. 10.1085/jgp.201110708 22851674 PMC3409101

[JCS191643C27] Terentyev D., Viatchenko-Karpinski S., Valdivia H. H., Escobar A. L. and Györke S. (2002). Luminal Ca2+ controls termination and refractory behavior of Ca2+-induced Ca2+ release in cardiac myocytes. *Circ. Res.* 91, 414-420. 10.1161/01.RES.0000032490.04207.BD 12215490

[JCS191643C28] Terentyev D., Viatchenko-Karpinski S., Gyorke I., Volpe P., Williams S. C. and Gyorke S. (2003). Calsequestrin determines the functional size and stability of cardiac intracellular calcium stores: Mechanism for hereditary arrhythmia. *Proc. Natl. Acad. Sci. USA* 100, 11759-11764. 10.1073/pnas.1932318100 13130076 PMC208831

[JCS191643C29] Wang W., Cleemann L., Jones L. R. and Morad M. (2000). Modulation of focal and global Ca^2+^ release in calsequestrin-overexpressing mouse cardiomyocytes. *J. Physiol.* 524, 399-414. 10.1111/j.1469-7793.2000.00399.x 10766921 PMC2269876

[JCS191643C30] Wei L., Hanna A. D., Beard N. A. and Dulhunty A. F. (2009). Unique isoform-specific properties of calsequestrin in the heart and skeletal muscle. *Cell Calcium* 45, 474-484. 10.1016/j.ceca.2009.03.006 19376574

[JCS191643C31] Zhang L., Kelley J., Schmeisser G., Kobayashi Y. and Jones L. (1997). Complex formation between junctin, triadin, calsequestrin, and the ryanodine receptor: proteins of the cardiac junctional sarcoplasmic reticulum membrane. *J. Biol. Chem.* 272, 23389-23397. 10.1074/jbc.272.37.23389 9287354

[JCS191643C32] Zissimopoulos S., West D. J., Williams A. J. and Lai F. A. (2006). Ryanodine receptor interaction with the SNARE-associated protein snapin. *J. Cell Sci.* 119, 2386-2397. 10.1242/jcs.02936 16723744

[JCS191643C33] Zissimopoulos S., Seifan S., Maxwell C., Williams A. J. and Lai F. A. (2012). Disparities in the association of the ryanodine receptor and the FK506-binding proteins in mammalian heart. *J. Cell Sci.* 125, 1759-1769. 10.1242/jcs.098012 22328519

